# Synthesis of Ordered Mesoporous CuO/CeO_2_ Composite Frameworks as Anode Catalysts for Water Oxidation

**DOI:** 10.3390/nano5041971

**Published:** 2015-11-17

**Authors:** Vassiliki Ι. Markoulaki, Ioannis T. Papadas, Ioannis Kornarakis, Gerasimos S. Armatas

**Affiliations:** Department of Materials Science and Technology, University of Crete, Vassilika Vouton, Heraklion 71003, Greece; E-Mails: vassiamark@gmail.com (V.I.M.); jpapadas@gmail.com (I.T.P.); gkornarakis@materials.uoc.gr (I.K.)

**Keywords:** mesoporous materials, cerium oxide, nanocasting, nanostructured, water oxidation

## Abstract

Cerium-rich metal oxide materials have recently emerged as promising candidates for the photocatalytic oxygen evolution reaction (OER). In this article, we report the synthesis of ordered mesoporous CuO/CeO_2_ composite frameworks with different contents of copper(II) oxide and demonstrate their activity for photocatalytic O_2_ production via UV-Vis light-driven oxidation of water. Mesoporous CuO/CeO_2_ materials have been successfully prepared by a nanocasting route, using mesoporous silica as a rigid template. X-ray diffraction, electron transmission microscopy and N_2_ porosimetry characterization of the as-prepared products reveal a mesoporous structure composed of parallel arranged nanorods, with a large surface area and a narrow pore size distribution. The molecular structure and optical properties of the composite materials were investigated with Raman and UV-Vis/NIR diffuse reflectance spectroscopy. Catalytic results indicated that incorporation of CuO clusters in the CeO_2_ lattice improved the photochemical properties. As a result, the CuO/CeO_2_ composite catalyst containing ~38 wt % CuO reaches a high O_2_ evolution rate of ~19.6 µmol·h^−1^ (or 392 µmol·h^−1^·g^−1^) with an apparent quantum efficiency of 17.6% at λ = 365 ± 10 nm. This OER activity compares favorably with that obtained from the non-porous CuO/CeO_2_ counterpart (~1.3 µmol·h^−1^) and pure mesoporous CeO_2_ (~1 µmol·h^−1^).

## 1. Introduction

The oxygen evolution reaction (OER) is a key chemical process in various electrochemical devices, such as rechargeable metal-air batteries and solar fuels. However, OER kinetics hinder the electrochemical oxidation of water to oxygen (2H_2_O → 4H^+^ + 4e^−^ + O_2_) and, thus, the overall efficiency of water splitting. This is due to the energetic cost (activation energy and enthalpy of adsorption) required for the dissociation of HO^−^ species participating in the OER and the formation of O–O bonds [1,2]. Over the past few years, a diverse range of metal-oxide semiconductors has been synthesized and extensively studied as anode catalysts for oxygen gas production. Typical examples are precious metal oxides, such as RuO_2_ and IrO_2_ [3], perovskites, such as BiFeO_3_ [4] and SrTiO_3_ [5], and transition-metal oxides (including hydroxides), such as BiVO_4_ [6], CoO*_x_* [7], MnO*_x_* [8], FeO*_x_* [8] and WO_3_ [9]. Nevertheless, thus far, little work has been conducted on the synthesis of highly porous rare-earth metal oxides and the investigation of their OER performance. These materials, although showing low solar light absorption (*i.e.*, absorbing light in the UV region), have received special attention in the fields of photovoltaics and photocatalysis because of their excellent electrical conductivity, chemical stability and reversible redox activity [10,11].

In this work, we present the synthesis, structural characterization and OER photocatalytic properties of ordered mesoporous frameworks composed of cerium(IV) oxide and copper oxide (CuO) compounds. Nanostructured CeO_2_-based materials have drawn attention as promising catalysts for CO oxidation [12,13], water-gas shift reaction [14] and degradation of organic pollutants [15]. Moreover, as an n-type semiconductor (*E*_g_ ~ 3–3.2 eV) with strong redox capability, CeO_2_ has also been used as an active component in several energy storage and conversion systems, including solar cells [16], solid oxide fuel cells [17,18] and solar hydrogen evolution devices [19,20]. To produce the mesoporous binary CuO–CeO_2_ oxides, we used a hard templating technique. The templated synthesis via the nano-replication route appears to be a versatile method to build porous multicomponent metal oxide materials [21]. In general, this method involves infiltration of suitable metal precursors within the nanopores of a solid template (e.g., carbon or silica) and thermal decomposition/solidification at elevated temperature. The resulting mesoporous solids, left after removal of the template by chemical etching or calcination, feature a three-dimensional (3D) nanoscale porous structure with a regular size and shape imparted by the template pore morphology. The obtained CuO/CeO_2_ heterostructures possess a 3D open-pore structure with a large internal surface area and exhibit good performance in photocatalytic oxidation of water. Our catalytic results showed that the 38% CuO-loaded CeO_2_ catalyst affords much higher OER activity than the other mesoporous composites, as well as the non-porous CuO/CeO_2_ counterpart and pure mesoporous CeO_2_, giving an oxygen evolution rate of ~19.6 μmol·h^−1^ under UV-Vis light irradiation.

## 2. Experimental Section

### 2.1. Synthesis of Mesoporous Silica

Hexagonal mesoporous Santa Barbara Amorphous-15 (SBA-15) silica was prepared under hydrothermal treatment at 100 °C for 2 days, according to the method reported by Zhu *et al.* [22]. This procedure uses 3 wt % poly(vinyl alcohol) solution to improve mesopore connectivity. The silica template was characterized by X-ray diffraction (XRD) (Figure S1) and N_2_ physisorption (Figure S2) measurements. The SBA-15 material was pretreated in air at room temperature for 2 days to increase the hydrophilic nature of the pore surface.

### 2.2. Synthesis of Mesoporous CuO/CeO_2_ Composites

In a typical preparation of mesoporous CuO/CeO_2_ composites, 1.1 mmol of metal nitrates, Ce(NO_3_)_3_·6H_2_O (≥99.5%, Alfa Aesar, Karlsruhe, Germany) and Cu(NO_3_)_2_·5H_2_O (98%, Alfa Aesar, Karlsruhe, Germany), were pre-mixed with 0.15 g of SBA-15 silica in 1.5 mL of hexane. The resulting viscous mixture was ground with an agate mortar for about 30 min to yield a fine paste, dispersed in 5 mL of hexane (≥99.5%, Sigma-Aldrich, Darmstadt, Germany) and, subsequently, stirred for 12 h under reflux at 70 °C. The solid product was isolated by filtration, washed with hexane and dried at 70 °C for 12 h. The resulting powder was then heated to 500 °C (2 deg·min^−1^ ramping rate) for 5 h to decompose the metal nitrate precursors. Finally, the silica matrix was selectively removed by treating two times with 2 M NaOH solution at room temperature for 2 h each time. The amount of Cu(NO_3_)_2_·5H_2_O used in the reactions was varied between 100, 132, 185 and 205 mg to gives a series of mesoporous CuO(*x*)/CeO_2_ materials with a different loading amount of CuO, *i.e.*, *x* ~ 16, 26, 38 and 45 wt %, respectively, according to the EDS analysis. For the comparison, mesoporous CeO_2_ (denoted as *mp*-CeO_2_) was prepared following a similar procedure, but using dried (at 150 °C for 3 h) mesoporous SBA-15 silica as the template and without the addition of copper(II) nitrate. Nonporous CuO/CeO_2_ composite with ~38 wt % of CuO content (denoted as *b*-CuO(38)/CeO_2_) was also prepared by direct calcination (500 °C, 5 h) of copper(II) and cerium(III) nitrate salts as the reference material.

### 2.3. Physical Characterization

The XRD patterns were collected on a PANalytical X´Pert Pro MPD X-ray diffractometer operated at 45 kV and 40 mA using Cu Kα radiation (λ = 1.5406 Å) in the Bragg–Brentano geometry. Nitrogen adsorption–desorption isotherms were measured at liquid N_2_ temperature (77 K) on a NOVA 3200*e* volumetric analyzer (Quantachrome, Boynton Beach, FL, USA). Before analysis, samples were degassed overnight at 150 °C under vacuum (<10^−5^ Torr) to remove moisture. The specific surface areas were calculated using the Brunauer–Emmett–Teller (BET) method [23] on the adsorption data in the 0.06–0.25 relative pressure (P/P_o_) range. The total pore volumes were derived from the adsorbed volume at P/P_o_ = 0.99, and the pore size distributions were obtained by the nonlocal density functional theory (NLDFT) method [24] based on the adsorption data. Elemental microprobe analyses were performed using a JEOL scanning electron microscopy (SEM) system (Model JSM-6390LV, Tokyo, Japan) equipped with an Oxford INCA PentaFETx3 energy-dispersive X-ray spectroscopy (EDS) detector (Oxfordshire, UK). Data acquisition was performed at least four times for each sample using an accelerating voltage of 20 kV and a 100-s accumulation time. Transmission electron microscopy (TEM) was performed using a JEOL Model JEM-2100 (Tokyo, Japan) electron microscope (LaB_6_ filament) operated at an accelerating voltage of 200 kV. Samples were prepared by sonicating the finely-ground powder in ethanol and depositing a drop of the resulting mixture onto a Cu grid covered with carbon film. Raman spectroscopy was performed at room temperature using a Nicolet Almega XR micro-Raman spectrometer (Thermo Scientific, Hudson, NH, USA) equipped with a 473 nm blue laser (15 mW) as an excitation source. UV-Vis/near-IR diffuse reflectance spectra were obtained on a Perkin Elmer Lambda 950 optical spectrophotometer (Baesweiler, Germany), using an integrating sphere. BaSO_4_ powder was used as a reflectance standard (100%), and the reflectance data were converted to absorption using the Kubelka–Munk equation: *a*/*S* = (1 − *R*)^2^/(2*R*), where *R* is the reflectance and *a* and *S* are the absorption and scattering coefficients, respectively.

### 2.4. Photocatalytic OER Reactions

The photocatalytic water oxidation reactions were carried out in a water-cooled (20 ± 2 °C) quartz reaction vessel (100 mL) using a 300-W Xe lamp (Variac Cermax, Wiesbaden, Germany). In a typical experiment, 50 mg of the catalyst were dispersed, with stirring, in 50 mL of aqueous solution containing 0.1 M NaOH and 0.02 M Na_2_S_2_O_8_. The reaction mixture was first purged with Ar flow for at least 40 min to ensure complete air removal and then irradiated with UV-Vis light (λ > 360 nm). The produced O_2_ was analyzed by gas chromatography (Shimadzu GC-2014, TCD detector, Ar carrier gas, Kyoto, Japan). In our studies, all of the examined catalysts yielded a stable colloidal dispersion in water, possibly due to the hydrophilic surface and small size of the particles; typical SEM images showed small agglomerates consisting of several primary particles with sizes of ~0.4–0.5 μm for *mp*-CeO_2_ and ~0.3 μm for CuO(38)/CeO_2_ (Figure S3).

For the quantum efficiency and UV-Vis photon conversion efficiency calculations, the average intensity of incident light was measured using a StarLite power meter equipped with a FL400A-BB-50 fan-cooled thermal sensor (Ophir Optronics Ltd, Jerusalem, Israel). The average intensity of irradiation was measured to be 41.6 mW·cm^−2^ using a bandpass filter of λ = 365 ± 10 nm (Asahi Spectra, Tokyo, Japan), and the intensity of irradiation in the wavelength range 360–780 nm was measured as 0.51 W·cm^−2^.

## 3. Results and Discussion

### 3.1. Morphology and Structural Properties

The chemical composition of CuO/CeO_2_ mesoporous materials was determined by energy dispersive X-ray spectroscopy (EDS). The EDS analysis of the different samples showed the presence of Ce and Cu in an atomic ratio from 0.56 to 2.45, which corresponds to ~16, ~26, ~38 and ~45 wt % of CuO loading; see Table 1. Note here that the CuO contents, as obtained by EDS, are consistently slightly lower than those expected from the stoichiometry of reactions (by 2–3 wt %) probably due to the insufficient infiltration of copper nitrate compounds into the silica template and dissolution of the CuO particles during the template removal process. All of the EDS spectra also showed a weak signal from Si, which is consistent with less than 5 wt % of the SiO_2_ residue remaining in products.

**Figure 1:**
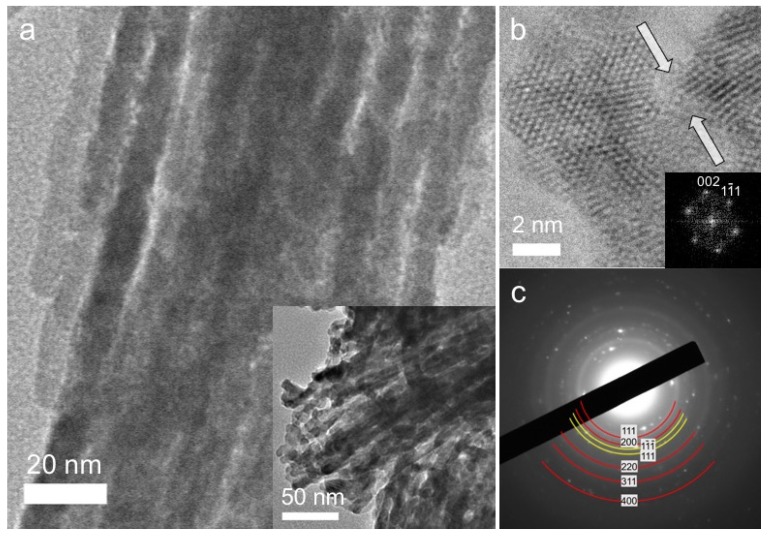
(**a**) Typical transmission electron microscopy (TEM) images; (**b**) High-resolution TEM image (the inset shows the corresponding FFT pattern indexed as the (110) zone axis of cubic CeO_2_) and (**c**) Selected-area electron diffraction (SAED) pattern of the mesoporous CuO(38)/CeO_2_ material. In (**b**), the white arrowheads indicate the bridge region between neighboring nanorods.

The mesoporous structure of the templated materials was investigated with transmission electron microscopy (TEM) and X-ray diffraction (XRD). Typical TEM images of the mesoporous CuO(38)/CeO_2_ sample are provided in Figure 1a. It can be seen that the CuO(38)/CeO_2_ mesostructure is constructed of uniform nanorods parallel to each other, consistent with the (110) direction of the hexagonal structure of the silica template. On the basis of the TEM analysis, the average diameter of nanorods was found to be ~8 nm, which is fairly close to the pore diameter of the silica template (~9.8 nm; Figure S2), indicating good replication of the silica mesostructure. For the investigation of the crystal structure of CuO(38)/CeO_2_, high-resolution TEM (HRTEM) images and selected-area electron diffraction (SAED) pattern were reordered. HRTEM taken from a thin area of the mesoporous framework shows well-resolved lattice fringes of (002) and ($1\bar{1}1$) planes of CeO_2_ with a *d*-spacing of 2.7 Å and 3.1 Å, respectively (Figure 1b). The image also shows connecting bridges between the nanorods, which attest to the structural coherence of the porous product. Figure 1c shows the typical SAED pattern of the mesoporous CuO(38)/CeO_2_. The SAED pattern depicts several Debye–Scherrer diffraction rings that can be indexed to the crystal planes of the CeO_2_ fluorite-type structure (marker with red lines) and the monoclinic lattice of CuO (marker with yellow lines).

**Table 1:** Analytical data and textural properties of mesoporous CeO_2_ (*mp*-CeO_2_) and CuO/CeO_2_ composite materials.

Sample	Atomic Ratio ^a^ (Ce:Cu)	CuO Loading (wt %)	Surface Area (m^2^·g^−1^)	Pore Volume (cm^3^·g^−1^)	Pore Size (nm)	Crystal Size ^b^ (nm)	Energy Gap (eV)
*mp*-CeO_2_			142	0.21	4.1	5.7	3.14
CuO(16)/CeO_2_	71:29	16	164	0.25	4.2	3.4	3.03
CuO(26)/CeO_2_	56:44	26	150	0.24	4.6	3.2	2.83
CuO(38)/CeO_2_	43:57	38	135	0.20	4.7	3.3	1.51
CuO(45)/CeO_2_	36:64	45	83	0.17	4.8	3.2	1.50

^a^ Based on the energy dispersive X-ray spectroscopy (EDS) analysis; ^b^ CeO_2_ crystallite size based on the Scherrer equation *D* = 0.9λ/βcosθ, where λ is the wavelength of the X-rays and β is the width (full-width at half-maximum) of the X-ray diffraction peak centering at a 2θ angle.

**Figure 2:**
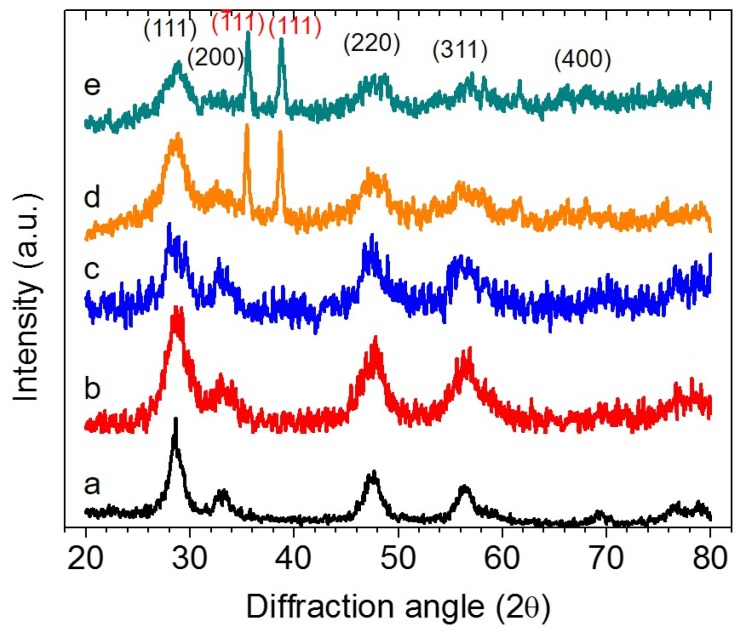
X-ray diffraction (XRD) patterns of mesoporous (**a**) *mp*-CeO_2_; (**b**) CuO(16)/CeO_2_; (**c**) CuO(26)/CeO_2_; (**d**) CuO(38)/CeO_2_ and (**e**) CuO(45)/CeO_2_ materials.

Figure 2 presents wide-angle XRD patterns of the mesoporous *mp*-CeO_2_ and CuO/CeO_2_ materials. It is proven that the nanocast products comprise crystallites of CeO_2_ and CuO with a small grain size. All of the XRD patterns display several broad Bragg diffraction peaks that can be readily indexed as (111), (200), (220), (311) and (400) diffractions of CeO_2_ with a fluorite-type structure (JCPDF No. 34–0394). The average domain size of the CeO_2_ crystallites calculated from Scherrer’s equation [25] and the broadening of the primary (111) peak is about 5 nm for *mp*-CeO_2_ and 3–4 nm for the CuO/CeO_2_ composites. The reduction in crystallite size for composite materials can be attributed to the inhibited grain growth of CeO_2_ during the conversion process due to the presence of CuO particles; such particles may be located near the grain boundaries, resulting in the formation of nanostructured CeO_2_ islands. In addition to CeO_2_ diffractions, the XRD patterns of CuO(38)/CeO_2_ and CuO(45)/CeO_2_ materials show intense peaks at ~35.5° ($\bar{1}11$) and ~38.7° (111) due to the crystalline phase of CuO (JCPDS No. 5-0661; space group: C2/c), indicating that copper(II) oxide species are growth within the CeO_2_ matrix. The structural assignment based on XRD is also collaborated by TEM experiments. The fact that CuO reflections in the XRD profiles of low CuO-loaded samples (CuO(16)/CeO_2_ and CuO(26)/CeO_2_) are not visible can be explained by the small grain size and high dispersion of CuO clusters in the ceria lattice.

**Figure 3:**
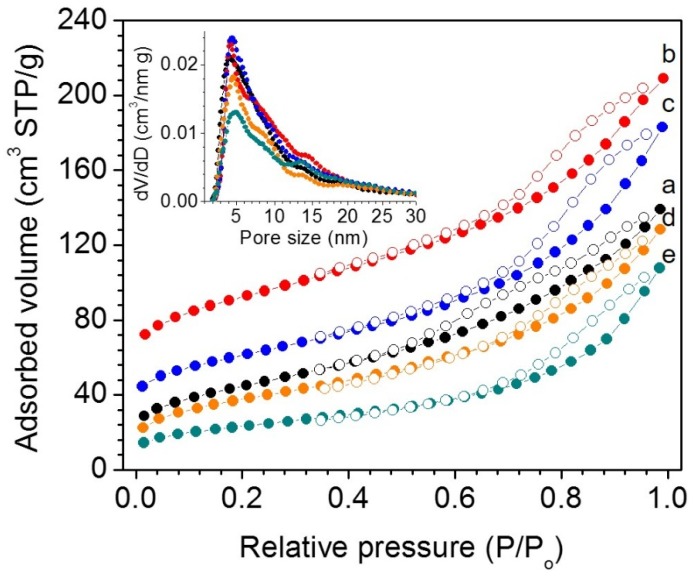
Nitrogen adsorption–desorption isotherms at 77 K and the corresponding nonlocal density functional theory (NLDFT) pore-size distribution plots calculated from the adsorption branch (inset) for mesoporous (**a**) *mp*-CeO_2_; (**b**) CuO(16)/CeO_2_; (**c**) CuO(26)/CeO_2_; (**d**) CuO(38)/CeO_2_ and (**e**) CuO(45)/CeO_2_ materials (STP: standard temperature and pressure). For clarity, the isotherms of (**a**), (**b**) and (**c**) are offset by 5, 40 and 20 cm^3^·g^−1^, respectively.

To evaluate porosity, the surface area and pore size of as-prepared materials were determined using N_2_ physisorption at 77 K. Figure 3 shows the N_2_ adsorption–desorption isotherms and the corresponding pore size distribution plots for mesoporous *mp*-CeO_2_ and CuO/CeO_2_ materials. All isotherms exhibit typical type-IV curves with an H_3_-type hysteresis loop, suggesting mesoporous structures with slit-like pores [26]. The mesoporous CuO/CeO_2_ composites were found to have Brunauer–Emmett–Teller (BET) surface areas in the range of 83–164 m^2^·g^−1^ and total pore volumes in the range of 0.17–0.25 cm^3^·g^−1^, while the mesoporous *mp*-CeO_2_ exhibited a surface area of 142 m^2^·g^−1^ and a total pore volume of 0.21 cm^3^·g^−1^. For composite samples with a moderate amount of CuO (less than 26 wt %), the increase in the surface area with the increase of CuO content could be attributed to the lower mass density of incorporated CuO (6.3 g·cm^−3^) relative to CeO_2_ (7.2 g·cm^−3^). As for the high CuO-loaded sample, CuO(45)/CeO_2_, the reduction of surface area and pore volume can be interpreted as a partial destruction of the pore structure. The pore width in as-prepared materials was determined by using the pore size analysis of the nonlocal density functional theory (NLDFT) adsorption model for slit-shaped pores and was found to be ~4–5 nm (inset of Figure 3). This pore size reflects the void space between the interconnected nanorods. The broad shoulder at 9–11 nm associated the pore size distributions corresponds to the large voids between the partially-interconnected nanorods. Table 1 summarizes the textural properties of mesoporous *mp*-CeO_2_ and CuO/CeO_2_ composite materials.

**Figure 4:**
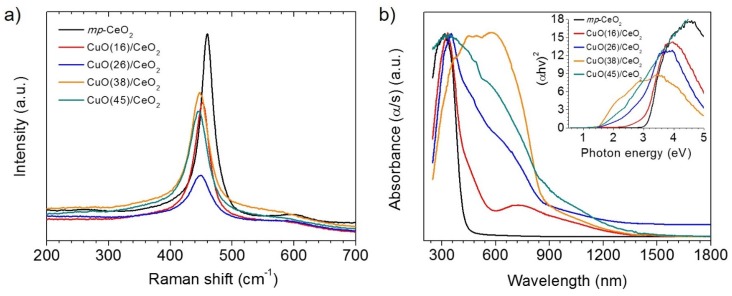
(**a**) Raman spectra and (**b**) ultraviolet-visible/near-IR (UV-Vis/NIR) diffuse reflectance spectra for mesoporous *mp*-CeO_2_ and CuO/CeO_2_ composite samples. Inset of (**b**) is the corresponding (α*hv*)^2^ *versus* energy curves, where α is the absorption coefficient, *h* is Planck’s constant and *v* is the light frequency.

In order to investigate the molecular and electronic structure of these CuO/CeO_2_ composites, we performed Raman and diffuse reflectance ultraviolet-visible/near-IR (UV-Vis/NIR) spectroscopy analysis. Raman spectroscopy is an intriguing tool to probe the crystal structure of oxide materials. The Raman spectra of *mp*-CeO_2_ and CuO/CeO_2_ materials, shown in Figure 4a, display an intense peak in the 445–459 cm^−1^ region that corresponds to the symmetrical stretching mode of {CeO_8_} units present in the CeO_2_. Compared to the Raman spectrum of *mp*-CeO_2_, the Ce–O absorption peak of composite samples shifts slightly to lower wavenumbers and becomes wider with increasing CuO content. This is due to the lattice distortion and the formation of framework defects (Ce^3+^ sites and oxygen vacancies) [27,28]. The broad band between 585 cm^−1^ and 600 cm^−1^ is associated with oxygen vacancies in the CeO_2_ lattice [29]. Evidence of CuO absorption was obtained from the Raman spectra of high CuO-loaded samples (*i.e.*, CuO(38)/CeO_2_ and CuO(45)/CeO_2_). The shift at 276 cm^−1^ in these spectra can be assigned to the one-phonon A_1g_ mode of CuO [30].

The UV-Vis/NIR absorption spectrum of *mp*-CeO_2_, transformed from the diffuse reflection data according to the Kubelka–Munk method, displays a sharp optical absorption edge at around 395 nm, which is associated with an energy gap at 3.14 eV (Figure 4b). This absorption is interpreted by the O_2p_ → Ce_4f_ electron transition in CeO_2_. Compared to *mp*-CeO_2_, the mesoporous CuO/CeO_2_ samples showed a significant red-shift in the absorption edge and absorb light in the visible region. In particular, the energy band gap of CuO/CeO_2_ varies systematically from 3.03 eV to 1.50 eV with increasing the CuO content. This behavior is probably due to the creation of oxygen vacancies in the CeO_2_ lattice, which allow the formation of the localized energy states between the O 2p (valence band) and Ce 4f states [31], as well as the incorporation of excessive CuO particles that absorb light in the near-infrared region of the spectrum (*E*_g_ ~ 1.5 eV).

### 3.2. Photocatalytic OER Reactions

**Figure 5:**
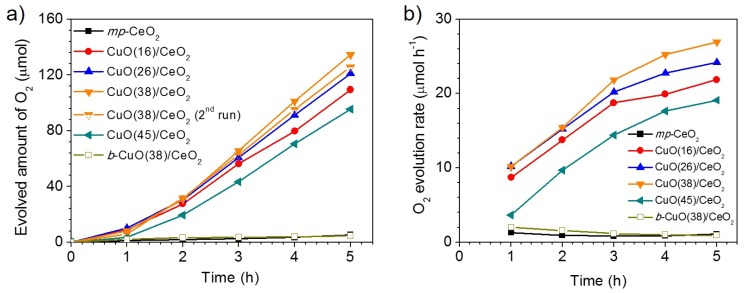
(**a**) Oxygen evolution curves and (**b**) time courses of photocatalytic O_2_ evolution rates for mesoporous *mp*-CeO_2_ and CuO/CeO_2_ composite materials and bulk *b*-CuO(38)/CeO_2_ solid.

We assessed the OER properties of CuO/CeO_2_ mesoporous materials in the UV-Vis light (λ > 360 nm)-irradiated oxidation of water using S_2_O_8_^2−^ as the sacrificial electron acceptor. Similar OER measurements for the pure mesoporous CeO_2_ (*mp*-CeO_2_) and non-templated *b*-CuO(38)/CeO_2_ samples were also performed for comparison. The oxygen evolution data in Figure 5a indicate that the mesoporous CuO/CeO_2_ composite containing a CuO content of ~38 wt % achieves higher OER activity than other examined catalysts, presenting an average O_2_ evolution rate of ~19.6 µmol·h^−1^ (or 392 µmol·h^−1^·g^−1^) with an apparent quantum efficiency (QE) of 17.6% at λ = 365 ± 10 nm and an incident photon conversion efficiency of around 1.3% under UV-visible light illumination (360–780 nm). The intrinsic OER activity of the examined catalysts was also examined by turnover frequency (TOF), assuming that every atom in the sample is involved in catalysis (Table 2). The mesoporous CuO(38)/CeO_2_ was found to exhibit the highest TOF of ~2.23 × 10^−5^ s^−^^1^. Remarkably, this OER activity represents a significant improvement compared to that of mesoporous *mp*-CeO_2_ (~1 µmol·h^−1^, TOF ~1.01 × 10^−6^ s^−1^), which indicates that the presence of CuO makes a significant contribution to enhancing the photocatalytic activity.

**Table 2:** Oxygen evolution reaction (OER) photocatalytic activity of the mesoporous *mp*-CeO_2_ and CuO/CeO_2_ materials and bulk *b*-CuO(38)/CeO_2_ solid.

Catalyst	O_2_ Evolution Rate (µmol·h^−1^)	Evolved O_2_, 5 h (µmol)	TOF ^a^ (s^−1^)
*mp*-CeO_2_	1.0	5.3	1.01 × 10^−6^
CuO(16)/CeO_2_	16.7	109.5	1.77 × 10^−5^
CuO(26)/CeO_2_	18.5	120.9	1.89 × 10^−5^
CuO(38)/CeO_2_	19.6 (0.9) ^b^	134.7	2.23 × 10^−5^
CuO(45)/CeO_2_	13.1	95.6	1.24 × 10^−5^
*b*-CuO(38)/CeO_2_	1.3	6.7	8.88 × 10^−7^

^a^ The turnover frequency (TOF) is defined as the number of O_2_ molecules produced per second per number of atoms in the catalyst; ^b^ Average O_2_ evolution rate under visible light irradiation (λ > 420 nm).

The kinetics of O_2_ evolution, shown in Figure 5b, indicate that the OER rate of composite samples increases over time, reaching, for example, a value of 26.9 µmol·h^−1^ after 5 h of illumination over the CuO(38)/CeO_2_ catalyst. This suggests the high propensity of the CuO/CeO_2_ materials to oxidize water. Control experiments revealed that no appreciable O_2_ evolution occurred when the reaction is conducted in dark or without catalyst or Na_2_S_2_O_8_, indicating that oxygen was produced by photocatalytic reactions. The greatly-improved OER activity of the CuO(38)/CeO_2_ catalyst is presumably a result of the synergistic action of CeO_2_ and CuO components. Specifically, we postulated that incorporation of CuO compounds within the CeO_2_ matrix can retard the recombination of photogenerated electrons and holes due to the interfacial electron transfer from CeO_2_ to CuO, thus enhancing photocatalytic performance. In addition, the introduction of CuO could be helpful for the formation of Ce^3+^ species (by the formation of framework oxygen defects) on the surface of CeO_2_; such species are recognized as active sites to enable OER activity [32]. Even through the incorporation of CuO particles in the CeO_2_ matrix can enhance the visible light absorption of CuO/CeO_2_ composites by introducing mid-gap states, it does not seem to be a reliable explanation for the increased photoactivity. As we will show, the CuO/CeO_2_ materials exhibit little photocatalytic activity under visible light. Therefore, we can conclude that when the loading amount of CuO increased, more CuO–CeO_2_ junctions and oxygen vacancies are formed in the CeO_2_ structure, resulting in the spatial separation of electron-hole pairs and the increase of the OER activity. The presence of oxygen vacancies in the lattice of CeO_2_ was also confirmed by Raman spectroscopy. As for the low OER activity of the overloaded CuO(45)/CeO_2_ sample, the CuO particles presumably shield a large part of the available CeO_2_ surface and, thus, cause the decrease of photocatalytic efficiency.

**Scheme 1:**
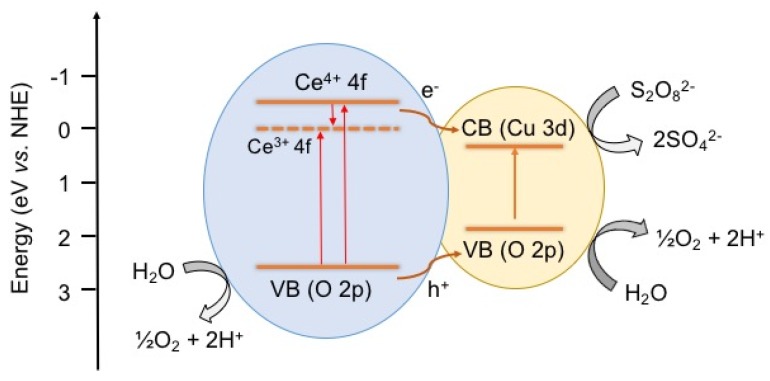
Photocatalytic O_2_ production mechanism on the CuO/CeO_2_ interface under UV-Vis light irradiation (VB: valence band, CB: conduction band, NHE: normal hydrogen electrode).

Photocatalytic water oxidation was also studied using visible light (λ > 420 nm) in order to examine if the CuO(38)/CeO_2_ catalyst works for visible energy conversion. It was found that CuO(38)/CeO_2_ exhibits an O_2_ evolution rate of ~0.9 µmol·h^−1^ under these conditions, which is much lower than that under UV-Vis light illumination. From the above observation, it appears that excitation of CuO alone (as CeO_2_ does not absorb light in the visible range) did not result in appropriate photocatalytic O_2_ production, and therefore, both CeO_2_ and CuO components are necessary for the CuO/CeO_2_ to absorb photons and trigger the photochemical process. Scheme 1 shows a schematic overview of the photocatalytic water oxidation reaction by mesoporous CuO/CeO_2_. First, photoexcited electrons (e^−^) and holes (h^+^) are generated, respectively, in the conduction band (CB) and valence band (VB) of both CeO_2_ and CuO under UV-Vis light illumination. Because of the potential gradient at the CuO–CeO_2_ interface, the photogenerated electrons on the Ce 4f orbitals of CeO_2_ can transfer to the CB of CuO and be consumed by the sacrificial agents S_2_O_8_^2−^; meanwhile, the photogenerated holes in the VB of CeO_2_ and CuO could effectively oxidize water to produce O_2_. As noted above, the Ce^3+^ cations in the CeO_2_ lattice (because of the presence of oxygen vacancies) may also act as electron-trap states to prevent the rapid recombination of photogenerated electrons and holes.

In addition, the superior OER activity of mesoporous CuO(38)/CeO_2_ could be attributed to the nanosized framework and highly accessible surface area, which favors easy diffusion of electrolytes. Evidence for this was obtained by comparing the OER activity of mesoporous CuO(38)/CeO_2_ to that of the non-templated, bulk counterpart (*b*-Cu(38)/CeO_2_). EDS, XRD and N_2_ physisorption measurements indicated that *b*-Cu(38)/CeO_2_ have similar composition and the same crystal structure as mesoporous CuO(38)/CeO_2_, but exhibit a low surface area (*ca*. 42 m^2^·g^−1^) (results not shown). As shown in Figure 5a, the non-templated *b*-Cu(38)/CeO_2_ catalyst exhibits poor photocatalytic OER activity with an average O_2_ evolution rate of ~1.3 µmol·h^−1^ over a 5-h period (TOF ~8.88 × 10^−7^ s^−1^), much lower than that of mesoporous analogues. Table 2 summarizes the OER activity data of the photocatalysts studied. We note that the OER activity of CuO(38)/CeO_2_ (~392 µmol·h^−1^·g^−1^) compares favorably with that obtained for Au/BiFeO_3_ nanowires (~380 µmol·h^−1^·g^−1^) [33], Pt/SrTiO_3_:Rh microparticles (~137 µmol·h^−1^·g^−1^) [34] and Au/CeO_2_ nanoparticles (~233 µmol·h^−1^·g^−1^) [35] and approaches that of the mesoporous Au/BiFeO_3_ heterostructure (~586 µmol·h^−1^·g^−1^) [4].

Moreover, the CuO(38)/CeO_2_ catalyst is stable and did not decompose during the photocatalytic process. As shown in Figure 5a, the CuO(38)/CeO_2_ manifested almost the same O_2_ production activity (within the experimental error) after two repeated runs. The total amount of O_2_ produced after 10 h of illumination is ~260.5 µmol (or ~5.8 mL), which is consistent with an average OER rate of ~26 µmol·h^−1^. Elemental X-ray microanalysis, X-ray diffraction and N_2_ adsorption–desorption isotherms confirmed that the reused catalyst maintains the chemical composition and textural properties of the fresh CuO(38)/CeO_2_ material. EDS results showed that the CuO content in reused sample was ~36 wt %, while the XRD data indicated the presence of cubic CeO_2_ and monoclinic CuO phases in the composite structure (Figure S4). The nitrogen adsorption isotherm evidenced no change in the mesoporous structure after 10 h of catalysis, showing a surface area of 131 m^2^·g^−1^ and a pore volume of 0.19 cm^3^·g^−1^ (Figure S5).

## 4. Conclusions

In summary, mesoporous CuO/CeO_2_ composite semiconductors have been successfully prepared via a nano-replication technique, using mesoporous silica (SBA-15) as a solid template. X-ray diffraction, high-resolution TEM and nitrogen physisorption measurements evidenced that the resultant materials are composed of a parallel arrangement of uniform nanorods and exhibit high BET surface areas and narrow-sized mesopores. The presence of CuO particles within the mesoporous matrix was confirmed by Raman spectroscopy, while the visible light response of CuO/CeO_2_ heterostructures was verified with diffuse reflectance UV-Vis/NIR spectroscopy. Catalytic results showed that mesoporous CuO/CeO_2_ materials are active catalysts for O_2_ production via UV-Vis light-driven water oxidation. At a CuO content of ~38 wt %, the CuO/CeO_2_ catalyst exerts a high O_2_ evolution rate of ~19.6 mol·h^−1^ (or 392 µmol·h^−1^·g^−1^) with a QE of 17.6% at λ = 365 ± 10 nm and incident photon conversion efficiency of 1.3% in the 360–780 nm range. The high OER activity is attributed to the presence of Ce^3+^ states (due to the oxygen vacancies) and CuO–CeO_2_ junctions that prevent rapid electron-hole recombination and the high specific surface area that promotes fast mass-transfer kinetics. These results demonstrate the potential of the CuO/CeO_2_ mesoporous heterostructures to serve as efficient and stable anodes for photocatalytic oxygen production.

## Supplementary Materials

Supplementary materials can be accessed at: http://www.mdpi.com/2079-4991/5/4/1971/s1.
