# Supplementary material for: Synthesis of Ordered Mesoporous CuO/CeO2 Composite Frameworks as Anode Catalysts for Water Oxidation
Source: Nanomaterials (Basel). 2015 Nov 17;5(4):1971–84. doi: 10.3390/nano5041971 (PMC5304801; doi:10.3390/nano5041971)
Supplement: Supplementary file 1 [file nanomaterials-05-01971-s001.pdf]

Supporting Information

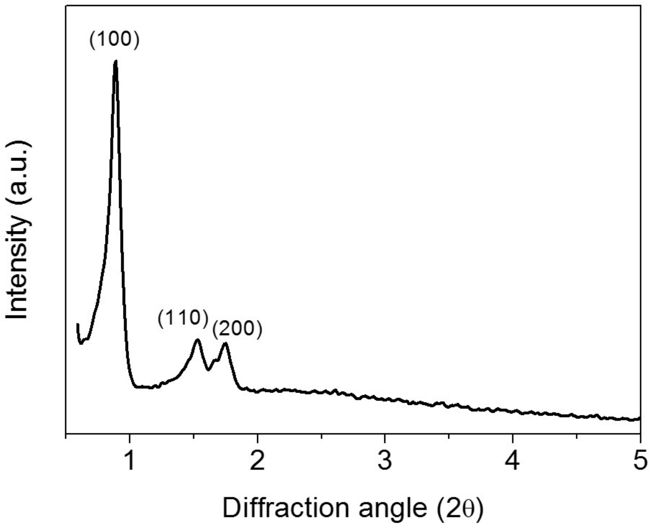

Figure S1. Low-angle X-ray diffraction (XRD) patterns of the mesoporous Santa Barbara Amorphous-15 (SBA-15) material. The angular position of the (100) Bragg diffraction is consistent with a hexagonal *p6mm* lattice parameter (a_o) of ~11.6 nm.

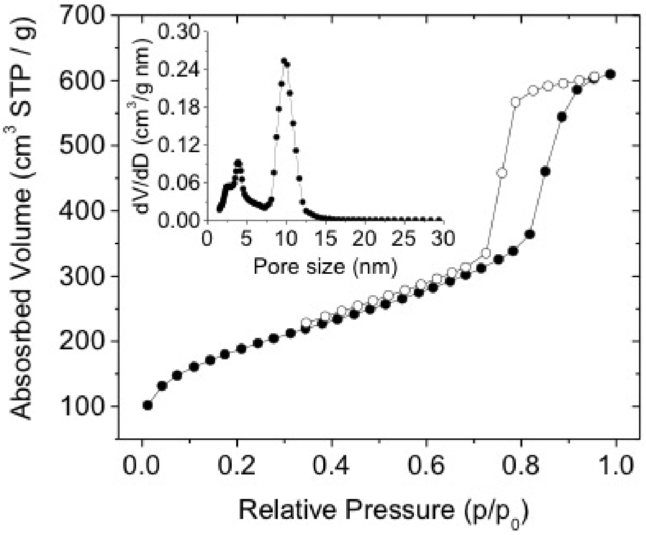

Figure S2. Nitrogen adsorption–desorption isotherms at 77 K and the corresponding nonlocal density functional theory (NLDFT) pore size distribution (inset) of the mesoporous SBA-15 material. The SBA-15 shows a specific surface area of 670 $m^2 \cdot g^{-1}$, a total pore volume of 0.95 $cm^3 \cdot g^{-1}$ and a narrow pore size distribution with a pore width of 9.8 nm.

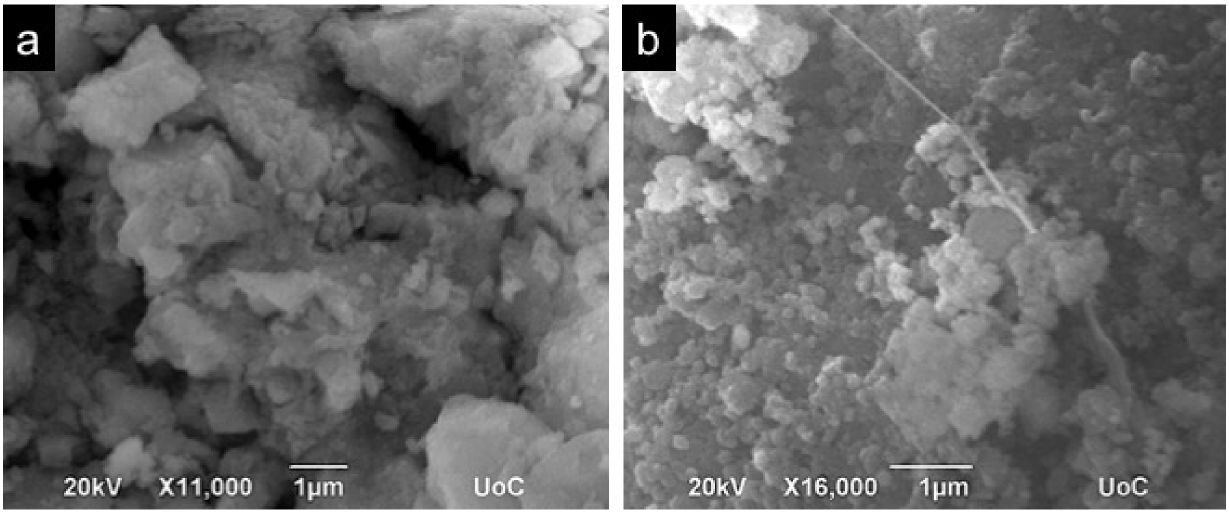

Figure S3. Typical scanning electron microscopy (SEM) images of mesoporous (**a**) *mp*-CeO_2 and (**b**) CeO(38)/CeO_2 materials.

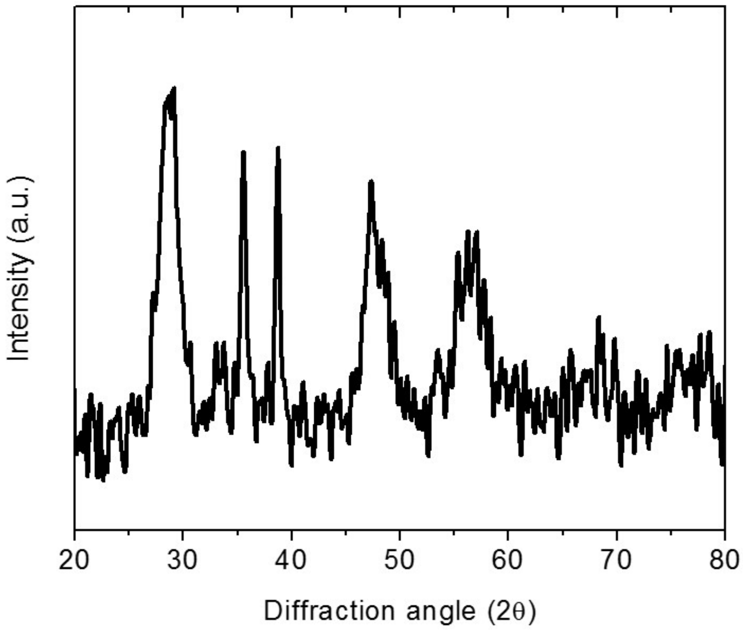

Figure S4. XRD pattern of reused CuO(38)/CeO_2 catalyst.

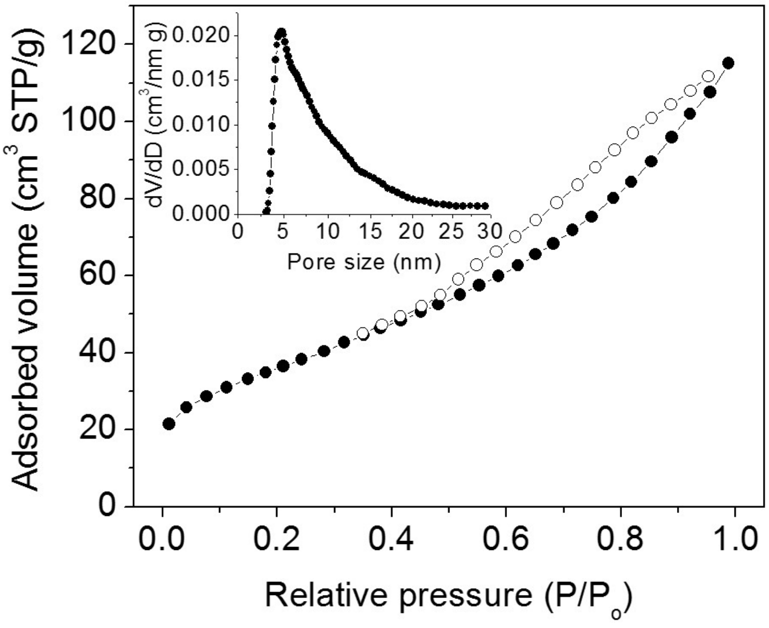

Figure S5. Nitrogen adsorption–desorption isotherms at 77 K of reused CuO(38)/CeO_2 catalyst. Analysis of the adsorption data with the Brunauer–Emmett–Teller (BET) method gives a surface area of 131 $m^2 \cdot g^{-1}$ and a total pore volume of 0.19 $cm^3 \cdot g^{-1}$. Inset: the corresponding NLDFT pore size distribution, indicating a pore size of ~4.8 nm.

© 2015 by the authors; licensee MDPI, Basel, Switzerland. This article is an open access article distributed under the terms and conditions of the Creative Commons Attribution license (http://creativecommons.org/licenses/by/4.0/).
